# Existence and functionality of emergency obstetric care services at district level in Kenya: theoretical coverage versus reality

**DOI:** 10.1186/1472-6963-13-113

**Published:** 2013-03-25

**Authors:** Elizabeth Echoka, Yeri Kombe, Dominique Dubourg, Anselimo Makokha, Bjørg Evjen-Olsen, Moses Mwangi, Jens Byskov, Øystein Evjen Olsen, Richard Mutisya

**Affiliations:** 1 Centre for Public Health Research Institute, Kenya Medical Research Institute (KEMRI), P.O. Box 20752–00202, Nairobi, Kenya; 2 Woman and Child Health Research Center, Department of Public Health, Institute of Tropical Medicine Nationalestraat 155, Antwerpen, 2000, Belgium; 3 Department of Food Science, Jomo Kenyatta University of Agriculture and Technology, PO Box 62000–00200, Nairobi, Kenya; 4 Centre for International Health, University of Bergen, PO Box 7804, Bergen, N-5020, Norway; 5 Department of Obstetrics and Gynaecology, Sørlandet Hospital, Flekkefjord, Norway; 6 Centre for Health Research and Development, Faculty of Health and Medical Sciences, University of Copenhagen, Thorvaldsensvej 57, Frederiksberg, DK 1871, Denmark

**Keywords:** Kenya, Maternal health, Emergency obstetric care, Life-saving

## Abstract

**Background:**

The knowledge on emergency obstetric care (EmOC) is limited in Kenya, where only partial data from sub-national studies exist. The EmOC process indicators have also not been integrated into routine health management information system to monitor progress in safe motherhood interventions both at national and lower levels of the health system. In a country with a high maternal mortality burden, the implication is that decision makers are unaware of the extent of need for life-saving care and, therefore, where to intervene. The objective of the study was to assess the actual existence and functionality of EmOC services at district level.

**Methods:**

This was a facility-based cross-sectional study. Data were collected from 40 health facilities offering delivery services in Malindi District, Kenya. Data presented are part of the “Response to accountable priority setting for trust in health systems” (REACT) study, in which EmOC was one of the service areas selected to assess fairness and legitimacy of priority setting in health care. The main outcome measures in this study were the number of facilities providing EmOC, their geographical distribution, and caesarean section rates in relation to World Health Organization (WHO) recommendations.

**Results:**

Among the 40 facilities assessed, 29 were government owned, seven were private and four were voluntary organisations. The ratio of EmOC facilities to population size was met (6.2/500,000), compared to the recommended 5/500,000. However, using the strict WHO definition, none of the facilities met the EmOC requirements, since assisted delivery, by vacuum or forceps was not provided in any facility. Rural–urban inequities in geographical distribution of facilities were observed. The facilities were not providing sufficient life-saving care as measured by caesarean section rates, which were below recommended levels (3.7% in 2008 and 4.5% in 2009). The rates were lower in the rural than in urban areas (2.1% vs. 6.8%; p < 0.001 ) in 2008 and (2.7% vs. 7.7%; p < 0.001) in 2009.

**Conclusions:**

The gaps in existence and functionality of EmOC services revealed in this study may point to the health system conditions contributing to lack of improvements in maternal survival in Kenya. As such, the findings bear considerable implications for policy and local priority setting.

## Background

Amid recognition that too many women die during pregnancy, childbirth and postpartum, the health system in Kenya continues to face challenges that may be contributing to lack of improvements in maternal survival. Recent estimates indicate that the maternal mortality ratio (MMR) remains unacceptably high, at 488 maternal deaths per 100,000 live births [1]. Kenya was also among the 11 countries contributing to 65% of all maternal deaths on a global scale in 2008, and one of the 23 countries in sub-Saharan Africa making insufficient progress towards Millennium Development Goal Five [2]. At this level of magnitude, improvements in maternal survival by 2015 present a key challenge.

There is evidence suggesting that most maternal deaths occurring in developing countries could be reduced if all women had access to interventions for treating complications that arise during pregnancy, childbirth and postpartum. This evidence reinforces the centrality of emergency obstetric care (EmOC) [3-10] in reducing maternal mortality. EmOC consist of a package of life-saving interventions or signal functions that include administration of parenteral antibiotics, uterotonic drugs, parenteral anticonvulsants, manual removal of placenta, removal of retained products of conception, assisted vaginal delivery by application of vacuum or forceps, neo-natal resuscitation, blood transfusion and caesarean section [10]. To describe the functionality and capacity of health systems in addressing life-threatening obstetric complications, a set of process indicators exist [10,11]. The indicators are based on the understanding that to reduce maternal deaths, obstetric services must be available and used by pregnant women. Table 1 shows the six EmOC process indicators issued in 1997 [11] with modifications on recommended level and the two new additional ones issued in 2009 [10].

**Table 1:** Emergency obstetric care indicators, questions and acceptable levels

**Indicator**	**Recommended level**
***Do the services exist and function?***	
1. Availability of EmOC: basic and comprehensive facilities	At least five EmOC facilities (including at least one comprehensive) per 500,000 population
***Are the services geographically and equitably distributed?***	
2. Geographical distribution of EmOC facilities	Equitably distributed in an area
***Are the services being used by pregnant women?***	
3. Proportion of all births in EmOC facilities	Recommended level set locally
***Are the services being used by women with complications?***	
4. Met need for EmOC services	100%
***Do they provide critical life saving services?***	
5. Caesarean section as a proportion of all births	5-15%
***Do they provide good quality care?***	
6. Direct obstetric case fatality rate	<1%
7. Intrapartum and very early neonatal death rate	To be set
8. Proportion of maternal deaths due to indirect causes	None set

Source: [10,11].

It is recommended that countries that intend to reduce maternal mortality should attempt to include EmOC process indicators into routine health management information systems to track progress in safe motherhood both nationally and at the lower levels of the health system [10,11]. However, the indicators are not routinely used in many countries. This includes Kenya, where only partial data from sub-national studies exist [12,13]. Although EmOC process indicators have been in existence since 1997, monitoring progress in maternal health goals in Kenya has relied heavily on the MMR, albeit the complexities surrounding this indicator [14-16]. Lack of sufficient information on obstetric care in Kenya may suggest that in a country with a high burden of maternal mortality, policy and decision makers are often unaware of the extent of need and, therefore, where to intervene. Another challenge is that obstetric care in Kenya is presented as a “crude” coverage, not taking into account the actual care provided in the health facilities. On the Ministry of Health website, all health centres are ‘automatically’ classified as basic EmOC [17], meaning that on paper, coverage is considered “good”. There is need for countries to classify EmOC facilities after direct inspection. This provides a distinction between how a facility is supposed to function and the reality. The distinction provides policy and decision makers with information necessary to improve coverage of services that can prevent maternal mortality and morbidity.

This study aimed to determine the actual situation in terms of existence, functionality and provision of critical life-saving services [10]. Malindi is one of the districts with a high MMR in Kenya, estimated at 625 maternal deaths per 100,000 live births by the district’s statistics. The evidence that a majority of these deaths could be averted if women have access to EmOC calls for the need to provide information on what interventions are needed to reduce these deaths.

Data presented draws from the “Response to accountable priority setting for trust in health systems” (REACT) study, whose intervention aimed at improving equity and access to quality health care at district level in Kenya, Tanzania and Zambia [18]. EmOC was one of the service areas selected to assess fairness and legitimacy of priority setting in health care. The larger REACT study included a baseline assessment of conditions for fairness and participation (and thus legitimacy) of priority setting and other decision making in the district health services, including EmOC. This aimed to assess whether fairness and participation in decision making could have an influence on service output and outcome. Ideally, such changes were expected to emerge after an active promotion of the fairness conditions [18].

Findings from this study thus provide information to the district health management team on the actual availability of EmOC as opposed to theoretical coverage in the district. The findings may therefore indicate if there is need to improve priority setting processes, which influence decision making on how to achieve optimal coverage and access to life-saving obstetric services for pregnant women in the district.

## Methods

This was a facility-based cross-sectional survey, conducted between October and December 2010 in Malindi District, Kenya. The district is located in the southern coastal region, covering an area of 7, 792 square kilometers. Four divisions: Malindi, Langobaya, Marafa and Magarini constitute the district. The total population in the district was 400,514 people in 2009, with urban–rural distribution of 140,739 and 259,775 persons, respectively [19]. Malindi division has a higher population density than the other three divisions as it has favourable topographic features and economic factors affecting human settlement. Malindi town, which is located in Malindi Division, has been labeled “Little Italy”, with an estimated 3,000 Italian residents. The district has a total of 105 public and private health facilities [17]. Of these, 42 (40%) offer delivery services. The total fertility rate in the district was 4.8 children per woman of reproductive age and crude birth rate of 38.1/1000 [20].

All the 42 facilities (private and public) that offer delivery services in Malindi District were listed for inclusion in the study. Since it was feasible to study all the facilities listed, no sampling was done. Two facilities were, however, not reached due to bad road conditions.

Although there are a total of eight process indicators, the study focused on the first, second and fifth indicators, since the aim of the study was to describe the actual situation in terms of existence and functionality of EmOC and provision critical life saving services. The first indicator examined the availability of EmOC. This was measured by obtaining data on the number of facilities that perform the complete set of signal functions. A standard tool was used to interview the in-charge of maternity unit, whether the nine signal functions had been performed at least once during the previous three months (Yes/No) [10]. If any of the signal functions had not been performed, reasons were recorded. A review of facility registers to ascertain that the signal functions were performed was done. In addition, observations to indicate the availability of equipment and drugs were conducted.

A strict WHO definition of a basic EmOC facility is one that has performed all the first seven signal functions in the last three months. A comprehensive EmOC facility is one that has performed caesarean section and blood transfusion in addition to basic functions in the past three months. In some instances, a signal function such as assisted delivery, is not performed in some countries as a matter of policy. According to the WHO handbook of assessing EmOC, “If a signal function is systematically absent in a region, it is possible to use the designation comprehensive “minus one” or basic “minus one” as a temporary measure while policies are reviewed and programmatic interventions planned to remedy the lack” [10].

The second indicator examined equity in distribution of facilities. This was achieved through mapping of facilities to identify gaps in geographical distribution of services and acknowledge added barriers such as distance to facilities. Geographical coordinates of different facilities were collected using a handheld Geographical Positioning System (GPS) device (Garmin eTrex). The device automatically logged in longitude and latitude values. Facility name, administrative location and type of facility were keyed in the device. The GPS data were downloaded into a spreadsheet and mapped onto an administrative map within ArcGIS 9.3 software environment. The map contained data from the survey department, with the most up to date official administrative boundaries. Road infrastructure and key features like settlements and water bodies were overlaid with the administration boundaries data to produce base maps. The GPS data were analysed in relation to administrative locality of facilities. This facilitated identification of underserved areas and approximate distance as an independent indicator of limitation to access. The conditions of roads and various terrain barriers were not considered since the buffer tool assumes a straight line distance function that would mean in real-time land travel. The buffer proximity analysis provided the shortest distance it would take to reach the comprehensive care facility.

The fifth indicator assessed the provision of critical life saving services for pregnant women as measured by caesarean section rates in the district. To obtain this data, a form was completed for every woman who underwent a caesarean section to obtain information on the indications for the intervention, geographical origin of the women and outcome for mother and newborn. The data were collected retrospectively for the periods 1^st^ January 2008 to 31^st^ December 2009. The data, together with district population figures [19] were used to calculate caesarean section rates by division and rural–urban residence of the women. The differences in rates between urban and rural women were compared using Pearson’s Chi-square test of association. The strength of the association was estimated using odds ratios, with corresponding 95% confidence interval.

Approval to conduct this study was obtained from the Kenya Medical Research Institute’s Ethical Review Committee (Scientific Steering Committee Number 1808). Written permission was obtained from the Medical Officer of Health in the district prior to visiting the health facilities. All data have been maintained as confidential and no individuals will be identified in dissemination of findings.

## Results

### Existence and functionality of emergency obstetric care

Among the 40 facilities assessed, three were hospitals, five were health centres while 32 were dispensaries. In terms of institutional deliveries, the numbers were higher in the government and hospital level facilities. Table 2 presents institutional deliveries in 2009 by facility ownership and level in the district.

**Table 2:** Distribution of institutional deliveries by facility ownership and level in 2009 in Malindi District, Kenya

***Ownership Level***	***Government***	***Private***	***Voluntary***	**Total**
Hospital	2893	249	-	3142
Health center	779	141	-	920
Dispensary	1472	200	167	1839
**Total**	**5144**	**590**	**167**	**5901**

In terms of existence of services, five of the 40 facilities assessed qualified as EmOC “minus one” (performing all nine signal functions except assisted vaginal delivery) [10]. These were the government hospital and two private hospitals, which qualified as comprehensive EmOC. Two government health centers qualified as basic EmOC. Table 3 shows the ratio of EmOC facilities to population size in Malindi District in 2010. With regard to the WHO recommendations, there should at least be five EmOC facilities for 500,000 inhabitants, with at least one being comprehensive. In this respect, with three hospitals and two health centres being EmOC, the WHO recommendation was met. However, going by the strict WHO definition of an EmOC facility, none of these facilities qualified since assisted vaginal delivery was not provided.

**Table 3:** Ratio of emergency obstetric care facilities to population size in 2010, Malindi District, Kenya

	**Ratio of facilities to population**	**Minimum recommended level**
**Basic EmOC**	2.5/500 000	4/500 000
**Comprehensive EmOC**	3.7/500 000	1/500 000
**Overall**	**6.2/500 000**	**5/500 000**

**Figure 1:**
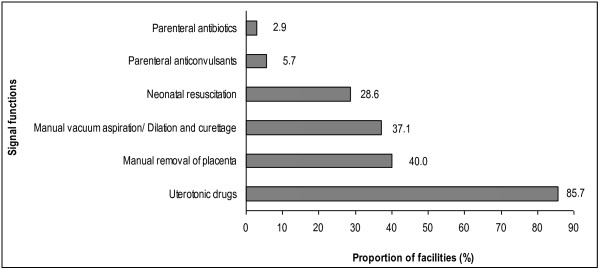
Contribution to health care of non emergency obstetric care facilities in providing basic signal functions in 2010, Malindi District, Kenya.

In terms of provision of signal functions, the least performed signal functions were administering parenteral antibiotics (2.9%) and parenteral anticonvulsants (5.7%). Uterotonic drugs (85.7%) and manual removal of placenta (40%) were most performed. Figure 1 shows the contribution to health care in provision of basic signal functions by the 35 non-EmOC facilities. The dominant reasons for least-performing signal functions were no cases and lack of supplies.

### Geographical distribution of facilities

**Figure 2:**
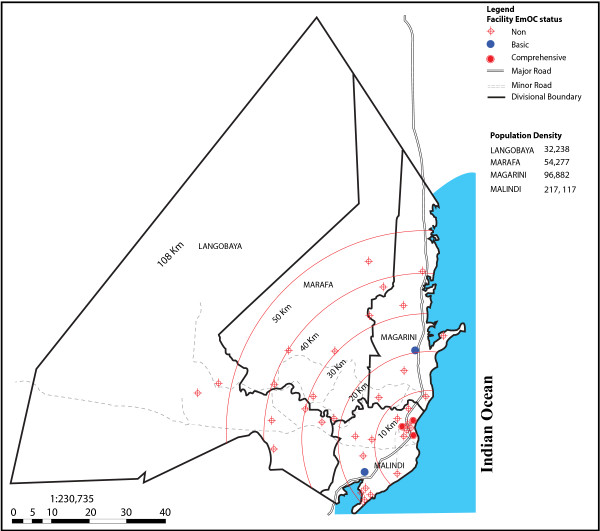
Distribution of delivery services by emergency obstetric care status in 2010 in Malindi District, Kenya.

Figure 2 shows the geographical inequities in distribution of EmOC facilities in the district. All the three hospitals offering comprehensive EmOC services and one of the two health centres offering basic EmOC services were located in Malindi Division, the main urban centre and administrative headquarters in the district. This area is served by a relatively well functioning public transport system and relatively adequate roads. The second basic EmOC facility was located in Magarini Division, which borders Malindi Division. This area is connected to the major trunk road with regular public transport. The two vast and remote divisions, Langobaya and Marafa, were not served by any EmOC facility and are not connected to any major trunk road with regular public transport. The average distance to the nearest EmOC facility was five kilometres in the urban area. Urban was defined as the area covered by 10 kilometres or less from the comprehensive EmOC facilities. For the rural areas, the average distance to the nearest comprehensive EmOC facility was 30 kilometres. Rural was defined as the area covered by more than 10 kilometres from the comprehensive EmOC facilities. Overall, Malindi Division had 19 facilities offering delivery services, Magarini had eight, Marafa had seven while Langobaya had six.

### Provision of sufficient life saving care: Caesarean section rates

A total of 539 and 687 forms were completed for women who underwent a caesarean section in the three comprehensive EmOC facilities in the district in 2008 and 2009 respectively. This amounted to over 90% of all surgical obstetric interventions performed in the hospitals in both 2008 and 2009. The most common indications for caesarean section were cephalopelvic disproportion and ante-partum haemorrhage. These accounted for 50.3% and 32% in 2008 and 54.6% and 27.7% in 2009, respectively.

The overall caesarean section rates were below the recommended level of 5%. These were 3.7% in 2008 and 4.5% in 2009. The rates were lower in the rural than in urban areas (2.1% vs. 6.8%; p < 0.001) in 2008 and (2.7% vs. 7.7%;p < 0.001) in 2009. Table 4 shows the caesarean section rates by division while Table 5 shows the rates by rural–urban residence in the district. A notable decrease in caesarean sections rates in divisions far from comprehensive EmOC facilities were observed.

**Table 4:** Caesarean section rates by divisions in Malindi District

	**Expected births**	**C-section performed**	**C-section rate**	**Estimated mean distance in kilometres to comprehensive EmOC facility**
**2008**				
**Division**				
Malindi	7534	436 (80.9%)	**5.8%**	5
Magarini	3578	84 (15.6%)	**2.3%**	10
Langobaya	1272	13 (2.4%)	**1.0%**	20
Marafa	2302	6 (1.1%)	**0.3%**	30
**Total**	**14,686**	**539**	**3.7%**	
**2009**				
**Division**				
Malindi	8272	541 (78.7%)	**6.5%**	5
Magarini	3691	105 (15.3%)	**2.8%**	10
Langobaya	1228	12 (1.7%)	**1.0%**	20
Marafa	2068	20 (2.9%)	**1.0%**	30
**Total**	**15,259**	**687**	**4.5%**	

**Table 5:** Caesarean section rates by rural and urban area of Malindi District

	**Expected births**	**N**^**o **^**of C-section performed**	**C-section rate**	**OR (95% CI); p-value**
**2008**				
Urban	4875	332	**6.8%**	3.39^* ^(2.83 – 4.06); **p < 0.001**
Rural	9811	207	**2.1%**	Reference
**Total**	**14,686**	**539**	**3.7%**	
**2009**				
Urban	5470	426	**7.8%**	3.17^*^ (2.70 – 3.72); **p < 0.001**
Rural	9789	261	**2.7%**	Reference
**Total**	**15,259**	**687**	**4.5%**	

^*^A pregnant woman with a complication requiring caesarean section in the urban area was up to 3 times more likely to access the intervention compared to a rural counterpart.

## Discussion

### Methodological issues and study limitations

This paper documents the application of EmOC process indicators to assess the actual existence, functionality and provision of life-saving obstetric services at district level in Kenya. Some limitations as well as methodological issues in the EmOC process indicators methodology were observed. The first limitation was related to the strict WHO categorisation of facilities as basic or comprehensive. This is based on the activities of a facility in the past three months. In this study, over 50% of facilities assessed had less than 60 deliveries in the previous three months. It is therefore not reasonable to presuppose that all seven signal functions would have been conducted within this period in these facilities. An extension of the period to six months would perhaps have given a chance to facilities that had the capacity to provide basic EmOC but did not qualify due to low deliveries. Secondly, the strict WHO criteria do not acknowledge the significant contribution to health care of facilities missing perhaps only one or two signal functions. Similar limitations in the EmOC process indicators methodology are documented elsewhere [21-23]. Thirdly, this paper is mainly descriptive and may not adequately provide a rigorous statistical appraisal of the findings. This limitation is however within the acceptable WHO concept of assessing the availability of EmOC [10]. Further, description of service level as provided in this paper provides a simple and clear message to decision makers and users and, therefore, no need for sophisticated statistical tests than necessary. Finally, the use of GIS buffer tool to estimate the distance women had to travel to reach the comprehensive care facility was a major limitation. The technique can be extremely misleading since the buffer tool assumes straight line distance, not taking into account road conditions and various terrain barriers.

### Existence and functionality of EmOC services

Health centres in Kenya are required to provide basic signal functions and are theoretically classified as basic EmOC facilities [17,24]. The Ministry of Health therefore ‘automatically’ classifies the five health centre level facilities in the district as basic EmOC [17]. The reality on the ground as evidenced in this study is however different. Only two of the five health centres expected to provide basic EmOC services qualified. A similarity illustrating theoretical coverage of facilities versus reality is provided in Uganda where, in 2003, results of an EmOC assessment showed that 21 (65%) of the 32 hospitals were comprehensive, while only five (4%) of the 129 Health centres functioned at their intended level [25]. The Uganda study is cited in the WHO handbook to show the difference between the way a facility is supposed to function and what it actually provides [10]. This highlights the need to classify facilities after direct inspection as opposed to theoretical coverage, which can be extremely misleading. In Kenya, by not taking the real definition of EmOC into account, EmOC coverage is often an overestimate.

The EmOC certification level requires that all signal functions are present [10]. Findings from this study, however, show that none of the facilities were “strictly" EmOC in the absence of assisted vaginal delivery by vacuum or forceps. Similar observations, showing that assisted vaginal delivery is a rare procedure, are documented in other sub-national studies in Kenya [12,13,26-28]. This is despite vacuum extraction being included in the minimum package for maternal health services at health centre and hospital level facilities [24,29]. Lack of the procedure implies that women who might benefit from this intervention are delivered by caesarean section. It is not clear why assisted vaginal delivery by vacuum is not offered as per the Ministry of Health and WHO specifications, yet there are observations that experienced midwives and nurses can perform the procedure at a basic EmOC facility [30]. In addition, anaesthesia is not required as with caesarean section and the potential risks associated with major haemorrhage and prolonged hospital stay are fewer [31]. Vacuum and forceps are also less invasive and inexpensive options in many situations [30]. Considering that comprehensive EmOC facilities are not easily accessible to rural women, vacuum extraction would be a more suitable option than caesarean section for women requiring a life-saving procedure in Malindi District and similar settings in Kenya. In Rwanda, a study revealed that under adequate supervision and immediate caesarean section back-up, vacuum deliveries conducted by competent mid-level providers are safe, making life-saving services more accessible and affordable for low-income households and rural populations [32].

The finding on lack of assisted vaginal delivery in this study and similar observations in other settings in Kenya highlight the need for further research to assess the status of the intervention in the country. This is necessary to provide information that may guide a review of maternal health guidelines to remedy the lack of this life-saving intervention in Kenya. Bridging this gap will contribute to strengthening the health system capacity to treat obstetric emergencies. There is also need to revisit the training policy on maternal health, given that midwives and clinical officers in Kenya are not currently trained to perform assisted vaginal delivery [33].

Comparable findings on availability of EmOC are reported in Kenya. The two Kenya Service Provision Assessment surveys of 2004 and 2010 found EmOC coverage to be below the recommended levels [12,13]. Similar to this study, adequate coverage in comprehensive EmOC facilities, but shortage of basic facilities was reported in North Eastern Province [26]. In the Nairobi’s informal settlements, among the facilities offering delivery services, only 40% were offering EmOC [27]. In West Pokot, although two facilities offered caesarean section and blood transfusion, no facility offered a complete set of signal functions in 2007 [28]. While the EmOC certification criteria is biased against facilities with capacity to provide EmOC but miss two or three signal functions perhaps due to few deliveries, their contribution should not be ignored. The analysis of signal functions by facilities that do not qualify the EmOC status as instituted in this study, not only illustrates the contribution to healthcare of these facilities, but also makes it possible to show which signal functions are not being performed adequately. This information can guide decision making on which areas require strengthening to achieve maximum gains in mortality and morbidity reduction. In this regard, the finding that signal functions were least performed due to low cases implies the need to determine whether it is because of low facility deliveries or if it a problem of access related issues, like costs and distance.

### Equity in geographical distribution of EmOC facilities

Equity in service provision, which entails fair distribution, access and use between population groups [34,35] is regarded as a measure of health system performance [36,37]. Although the optimal requirement of comprehensive EmOC facilities was met in this study, reaching them was a challenge for women in rural areas. While it is logical that such facilities are located in the urban areas [38,39] because of population density and presence of good infrastructure, it is clearly unfair to rural women. Except if compensated by an effective referral infrastructure that includes adequate good roads, communication and emergency transport. In view of the urgency and unpredictability of maternal complications, the inequities in access to care facilities in this study portray serious implications for women living in the underserved areas. These women have to travel over 30 kilometres to reach a comprehensive EmOC facility. Consequently, their chances of surviving an obstetric emergency are greatly reduced. Travel delay also means that the women arrive at the facility in such severe conditions that it may be difficult to save their lives [34]. If indeed policy and decision makers in countries with high maternal mortality are committed to the statement that "no woman should die while giving birth", there is need to improve equitable access to life saving interventions for women in under served areas. Geographical mapping of care facilities as instituted in this study can guide decision making on which facilities may benefit from upgrading to achieve the optimal coverage in an area [37,40]. Possibly, upgrading the health centre in Marafa Division to comprehensive and a dispensary in Langobaya Division to basic EmOC status or strengthening the referral infrastructure could contribute to fair distribution and access to obstetric care in the district. Upgrading should additionally involve providing the neccesary equipment and supplies to provide the services.

The relative lack of access to life-saving services for rural women as observed in this study is documented in other developing countries [21,40]. Similar, analysis of EmOC in countries with high and moderate levels of maternal mortality show that even as EmOC facilities meet the recommended levels, concerns surrounding equity and geographical accessibility are raised [40]. Similarly in this study, the question whether *EmOC services exist and function and if the services are geographically and equitably distributed* cannot be answered affirmatively for the rural areas.

### Need for critical life-saving services

Caesarean sections are regarded as the simplest ways of measuring the need for critical life-saving services [7,8,41]. In this study, the overall caesarean section rates in the district were below the recommended levels. Similar findings on low caesarean section rates are observed in other developing countries [21,27,42-46]. The differences in physical access to the comprehensive EmOC facility by women in the district undoubtedly explain the variations in caesarean section rates between the four divisions and urban–rural areas to a great extent. From the findings, a pregnant woman with a complication requiring caesarean section in the urban area was up to three times more likely to access the intervention compared to her rural counterpart. Perhaps by availing vacuum extraction, women with prolonged second stage of labor in the rural areas can benefit from the intervention instead of travelling long distances to access the comprehensive EmOC facilities. Findings on low caesarean section rates among women in the rural areas are reported in other studies in Kenya and Tanzania [44,47,48]. It is observed that caesarean section rates is a valuable process indicator for identifying gaps in obstetric care and may be used for advocating improvements for healthcare to the relevant authorities [42,47]. Thus, the variation between caesarean section rates in urban and rural areas observed in this study may indicate the need to upgrade some facilities to achieve good coverage in the rural areas. This alone, may however not increase use of life-saving services for rural women given that in this setting, the proportion of home deliveries is 84 percent (REACT data, unpublished). Sensitising the community to promptly seek care in the event of obstetric complications may be necessary to achieve optimal utilisation of life-saving services in the district.

## Conclusion

The EmOC process indicators methodology as applied in this study highlights the gaps in availability of services likely to reduce maternal mortality. The need to improve local priority setting and decision making towards achieving optimal coverage of life-saving obstetric interventions for pregnant women in the district is strongly recommended. The EmOC process indicators methodology may not have been entirely appropriate to assess availability of EmOC in a setting with low facility deliveries as Malindi District. As such, modifications to extend the three-month assessment period to six months may be required in similar settings. This will value the contribution to health care of facilities with capacity to provide EmOC, but which do not qualify due to few deliveries.

## Abbreviations

EmOC: Emergency Obstetric Care; GPS: Geographical Positioning System; MMR: Maternal Mortality Ratio; REACT: Response to Accountable priority setting for Trust in health systems; WHO: World Health Organisation.

## Competing interests

The authors declare that they have no competing interests.

## Authors’ contributions

EE was involved in the study design, data collection, analysis and initiated the manuscript. YK coordinated data collection and critically revised the manuscript. DD participated in the study conception, analysis and critically revised the manuscript. AM contributed substantially to writing and critically revising the manuscript. BEO co-coordinated the work package on EmOC and critically revised the manuscript. JB and OEO initiated and coordinated the overall “REACT” study and contributed substantially to editing of this manuscript. MM participated in data analysis and critically revised the manuscript. RM contributed to critically revising the manuscript. All authors read and approved the final manuscript.

## Pre-publication history

The pre-publication history for this paper can be accessed here:

http://www.biomedcentral.com/1472-6963/13/113/prepub
